# Effects of low-frequency ultrasound combined with anti-MRSA agents on the mouse model of pulmonary infection

**DOI:** 10.1128/spectrum.01016-23

**Published:** 2024-02-07

**Authors:** Kaicheng Yan, Jiahui Yao, Lingling Liu, Wenxin Liang, Yun Cai

**Affiliations:** 1 Department of Pharmacy, Center of Medicine Clinical Research, Medical Supplies Center, Chinese PLA General Hospital, Beijing, China; 2 Unit 32701 of Chinese PLA, Beijing, China; NHLS Tygerberg/Stellenbosch University, Cape Town, South Africa

**Keywords:** low-frequency ultrasound, pneumonia model, MRSA, antibiotics, distribution

## Abstract

The treatment of methicillin-resistant *Staphylococcus aureus* (MRSA)-induced pneumonia with antibiotics alone poses considerable challenges. To address these challenges, low-frequency ultrasound (LFU) emerges as a promising approach. In this study, a mouse pneumonia model was established through intratracheal injection of MRSA to investigate the therapeutic efficacy of LFU in combination with antibiotics. Minimal inhibitory concentration was assessed, and the distribution of antibiotics in the lung and plasma was determined using liquid chromatography coupled with mass spectrometry. Various parameters, including the survival rate, histopathology, lung bacterial clearance, and the expressions of cytokines and inflammation-related genes, were evaluated before and after treatment. Compared with the infection group, both the antibiotic-alone groups [vancomycin (VCM), linezolid, and contezolid (CZD)] and the groups in combination with LFU demonstrated an improvement in the survival status of mice. The average colony-forming units of lung tissue in the LFU combination groups were lower compared with the antibiotic-alone groups. While no significant changes in C-reactive protein and procalcitonin in plasma and bronchoalveolar lavage fluid were observed, histopathological results revealed reduced inflammatory damage in LFU combination groups. The secretion of interleukin-6 and tumor necrosis factor-alpha was decreased by the combination treatment, particularly in the VCM + LFU group. Furthermore, the expressions of MRSA virulence factors (*hla* and *agrA*) and inflammation-related genes (*Saa3*, *Cxcl9,* and *Orm1*) were further reduced by the combinations of LFU and antibiotics. Additionally, LFU treatment facilitated the distribution of VCM and CZD in mouse lung tissue at 30 and 45 min, respectively, after dosage.

**IMPORTANCE:**

Treating pneumonia caused by methicillin-resistant *Staphylococcus aureus* (MRSA) with antibiotics alone poses significant challenges. In this *in vivo* study, we present compelling evidence supporting the efficacy of low-frequency ultrasound (LFU) as a promising approach to overcome these obstacles. Our findings demonstrated that LFU enhanced the effectiveness of vancomycin, linezolid, and contezolid in an MRSA pneumonia model. The combination of LFU with anti-MRSA agents markedly improved the survival rate of mice, accelerated the clearance of pulmonary bacteria, reduced inflammatory injury, inhibited the production of MRSA endotoxin, and enhanced the distribution of antibiotics in lung tissue. The application of LFU in the treatment of pulmonary infections held substantial significance. We believe that readers of your journal will find this topic of considerable interest.

## INTRODUCTION

As a primary contributor to nosocomial pneumonia and healthcare-associated (HCA) pneumonia, methicillin-resistant *Staphylococcus aureus* (MRSA) is linked with significant morbidity and mortality (1, 2). MRSA is responsible for 10%–40% of cases of HCA pneumonia, hospital-acquired pneumonia (HAP), and ventilator-associated pneumonia (VAP) (3). MRSA pneumonia is characterized by a pronounced inflammatory reaction in the lungs, resulting in airway neutrophil influx, loss of alveolar structure, severe pulmonary edema, hemorrhage, and intrapulmonary bacterial proliferation. The mortality rate associated with MRSA HAP/VAP can reach as high as 55% (4), leading to prolonged intensive care unit (ICU) and hospital stays, extended mechanical ventilation duration, and increased costs in patients with MRSA pneumonia. Guidelines for nosocomial pneumonia recommend vancomycin (VCM) and linezolid (LZD) as empirical anti-MRSA therapies in at-risk patients (5). The recent elevation of minimum inhibitory concentration (MIC) with VCM has raised concerns about the use of other anti-MRSA drugs (6). It has been reported that the VCM concentration in the lungs is well below the MIC level needed for *S. aureus* at the current recommended dosage (7). Increasing the dosage of VCM poses a greater risk of nephrotoxicity (8). LZD has excellent activity against many important Gram-positive bacteria and is well distributed in lung tissue. However, the use of LZD is somewhat limited due to myelotoxicity when used long term (9). Contezolid (CZD) is a new oxazolidinone that exhibits potent activity against Gram-positive bacteria, including MRSA, methicillin-resistant *Streptococcus epidermidis*, penicillin-resistant *Streptococcus pneumoniae*, and VCM-resistant *enterococci* (10). Compared with LZD, a major potential advantage of CZD is its significantly improved safety profile with minimal myelosuppression and monoamine oxidase inhibition. At present, CZD is only approved for the treatment of complex skin and soft tissue infections. Overall, therapeutic options against MRSA pneumonia are still limited and lack clinically proven novel antimicrobials. Current antimicrobial therapy is indispensable in MRSA pneumonia treatment, but certain limitations, such as distribution in tissues, adverse events, and increased microbial resistance patterns, exist (11). Therefore, finding new ways to overcome obstacles in the treatment of pneumonia caused by MRSA is necessary.

Low-frequency ultrasound (LFU) refers to ultrasound with a frequency generally ranging from 20 to 200 kHz (12). Compared with high-frequency ultrasound, LFU is characterized by a lower frequency, longer wavelength, relatively less sound energy absorption, and higher power, making LFU easier to penetrate body tissue and causing less damage (13). LFU has various biological effects, such as mechanical, thermal, and cavitation effects (14). More attention has been given to the role of LFU in clinical treatment, such as promoting percutaneous penetration of medicines, accelerating coronary plaque ablation and thrombolysis, assisting in inhibiting tumor formation, and alleviating diabetic peripheral neuropathic pain (15–17). Clinical practice has shown that the benign nature of LFU energy is safe and feasible compared with other physical methods (18).

In the anti-infection field, physical LFU can not only enhance the anti-infection ability of antibiotics but also reduce the risk of bacterial antibiotic resistance (19). Other researchers and we have found that LFU can improve the antibacterial effect of various antibiotics on planktonic and biofilm bacteria *in vitro* and *in vivo*, such as MRSA, *Pseudomonas aeruginosa*, and *Klebsiella* pneumoniae (20–22). Here, our aim was to investigate the effect of LFU combined with anti-MRSA agents in a mouse pulmonary infection model caused by nosocomial MRSA via tracheal infection.

## RESULTS

### MICs for antimicrobial agents

The MIC values of VCM, LZD, and CZD to MRSA 159228 were 0.5, 2, and 1 µg/mL, respectively, indicating that MRSA 15928 was sensitive to VCM, LZD, and CZD.

### Mouse behavioral changes and survival rates

**Fig 1:**
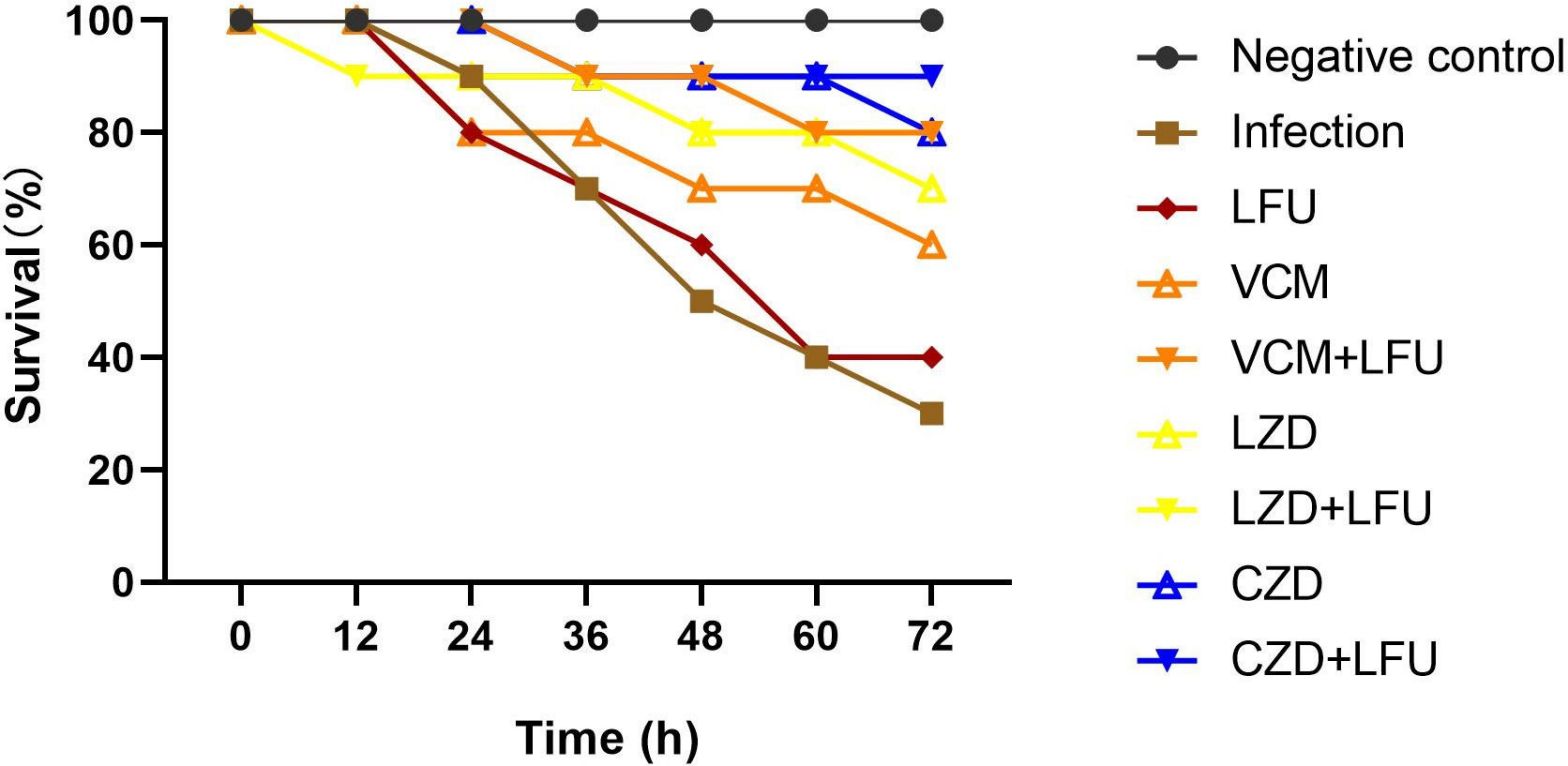
The survival rate of mice in each group (*n* = 10) was monitored 72 h after infection (60 h after treatment).

Before the MRSA 159228 infection, all mice exhibited smooth and shiny fur, along with a normal appetite. They were lively, active, and responsive to various external stimuli, such as sound and light. At 12 h after inoculation, mice displayed varying degrees of dry and matted fur, increased cheese-like secretion from the corners of the eyes, shortness of breath, dyspnea, reduced appetite, weight loss, mental depression, a curled-up posture, and an obvious slow response to the outside world. Except for the control group, animals in the other eight groups began to succumb to the infection from 12 h onward. By 72 h, the infection-related symptoms in mice from the infection group and the LFU group were the most severe, with survival rates of 30% and 40%, respectively (Fig. 1). Mice in these two groups did not drink water most of the time and appeared to be in a continuous underweight state with chills. In the VCM and LZD groups, mice occasionally drank water without evident shivering, and their mental state was better. Mice in the VCM + LFU, CZD, CZD +LFU, and LZD + LFU groups showed no obvious symptoms, and their physical conditions almost returned to a healthy state before infection. Compared with the VCM group at 60%, the survival rate of the VCM + LFU group increased to 80%. The survival rate of the LZD or CZD group vs their corresponding LFU combination groups was 70% vs 80% and 80% vs 90%, respectively. From the 60th hour onward, although there was no statistical difference in the survival rate between the LFU combined with the antibiotics group and the antibiotic-alone group, a significant difference was observed between the six treatment groups and the infection group.

### Lung bacterial load

**Fig 2:**
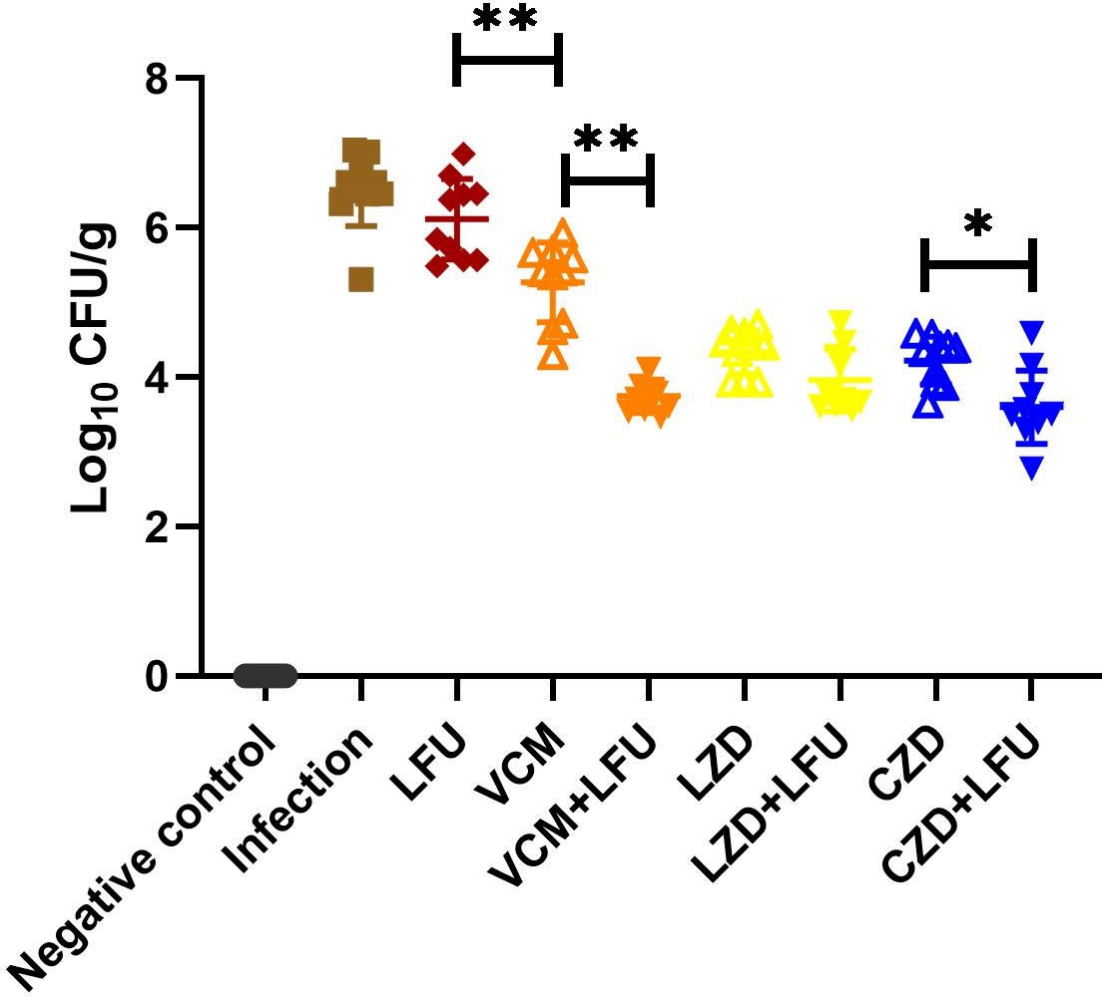
Lung bacterial clearance with three antibiotics and antibiotics + LFU in the MRSA-induced pneumonia model 54 h (6 h after the last treatment) after infection [means ± standard deviation (SD); *n* = 10; *P < 0.05; **P < 0.01].

Tissue homogenates from the right lung were collected at 54 h to assess bacterial load (Fig. 2). The average bacterial load in the infection group and the LFU group was elevated, measuring up to 6.3 log_10_ CFU/g. In contrast, the bacterial load in the antibiotic and antibiotic + LFU groups was significantly lower than that in the infection group. Specifically, the bacterial load in the VCM group was 5.3 log_10_ CFU/g, while in the VCM + LFU group, it was notably reduced to 3.7 log_10_ CFU/g. For the LZD and LZD + LFU groups, bacterial loads were similar at 4.3 and 3.9 log_10_ CFU/g, respectively. The bacterial load in the CZD + LFU group was lower than that in the CZD group (3.6 vs 4.2 log_10_ CFU/g). Notably, the bacterial load data were 0 in the negative control group, and there was a significant difference in bacterial loads between the infected and all treatment groups compared with the control group.

### Lung histopathology and injury scores

**Fig 3:**
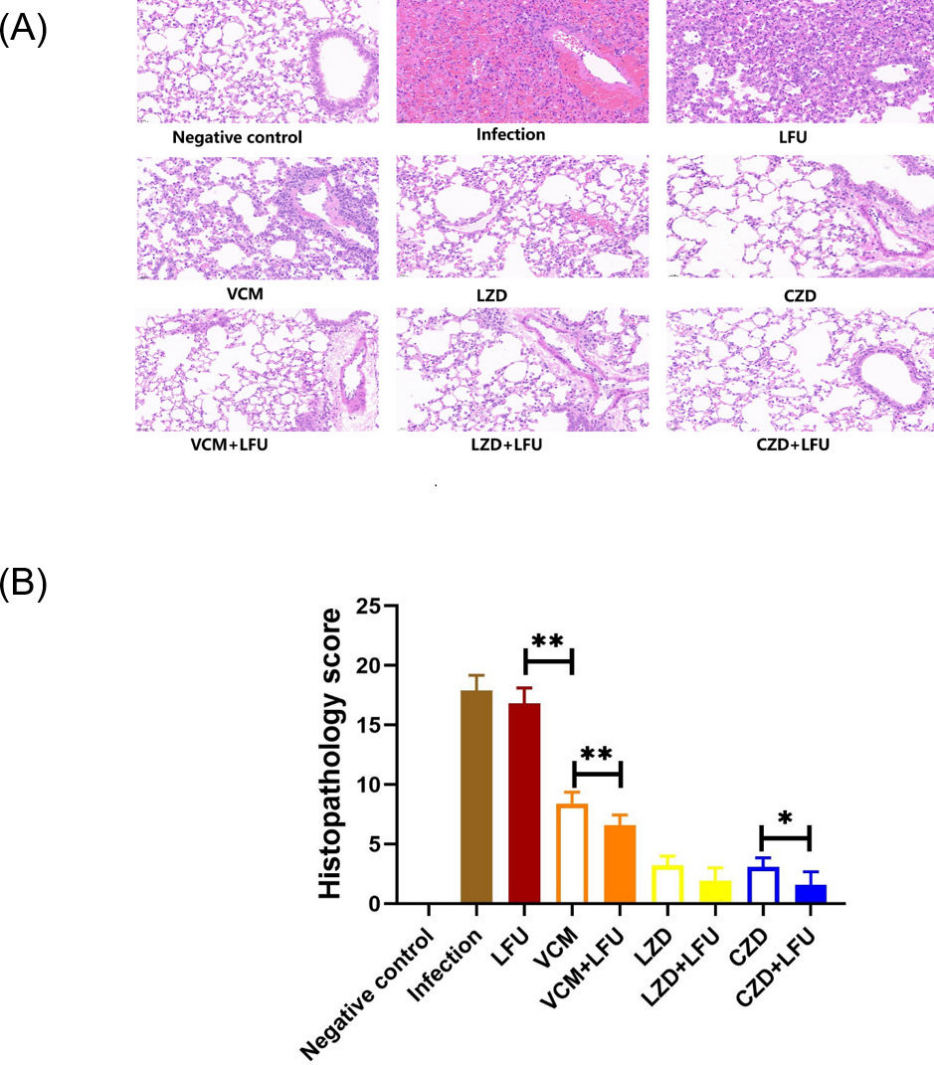
Lung histopathology and injury scores at 54 h (6 h after the last treatment) after infection. (**A**) Representative H&E stained sections of mouse left lung. (**B**) Histopathological injury scores [means ± standard deviation (SD); *n* = 10; *P < 0.05; **P < 0.01].

At 54 h, the left lungs of the mice were stained with hematoxylin/eosin (H&E) and observed under a microscope (Fig. 3A). In comparison with the negative control group, the infection group and the LFU group exhibited obvious infiltration of inflammatory cells, large-area necrosis of lung tissue, a significant presence of necrotic cell fragments, disintegration, destruction of the bronchial structure, and inflammatory cells in the bronchial lumen. Inflammation gradually eased in the antibiotic and antibiotic + LFU groups. Figure 3B illustrates that the average injury score of the infection group and the LFU group was 17.9 and 18.6, respectively (*P* > 0.05). The scores of all treatment groups were significantly lower compared with the infection group. CZD-containing groups displayed the lowest scores. The scores of the LFU combination groups were lower compared with their corresponding antibiotic-alone groups. VCM-containing groups showed the most significant decline.

### Cytokine concentration in plasma and bronchoalveolar lavage fluid

**Fig 4:**
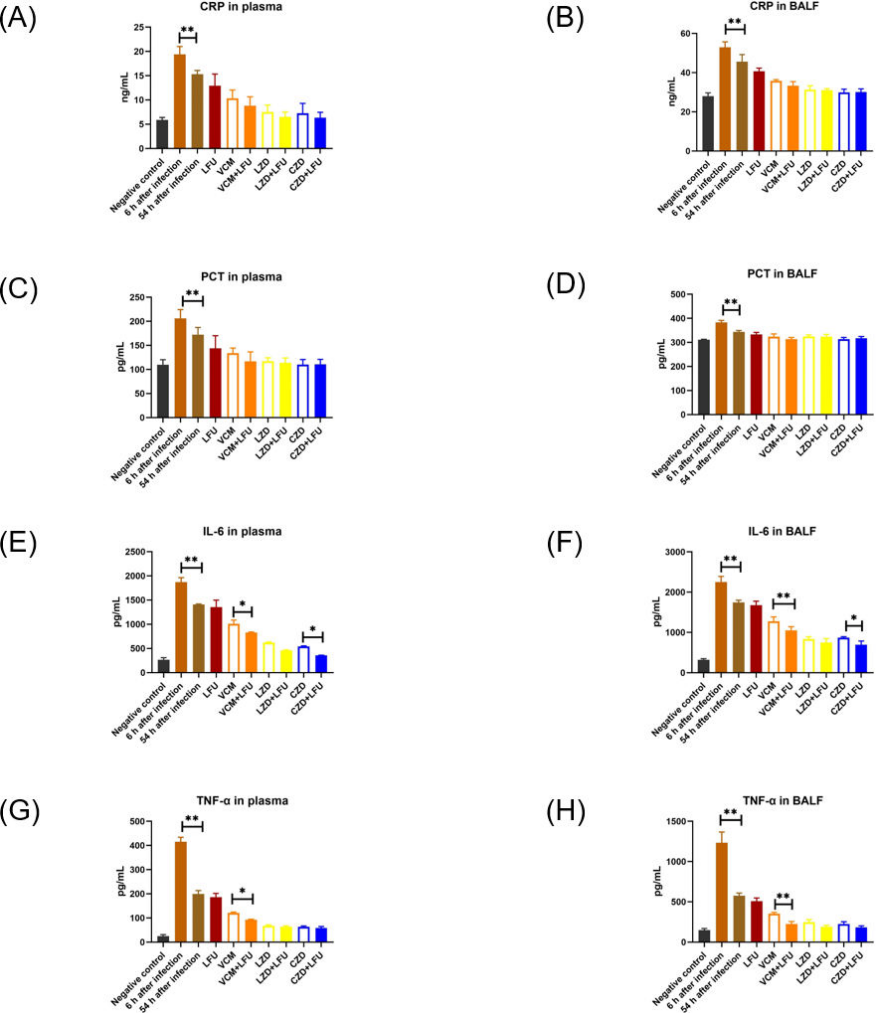
Cytokine concentrations (CRP, PCT, IL-6, and TNF-α) in plasma and BALF at 6 and 54 h (6 h after the last treatment) after infection. (A-D) The concentrations of CRP and PCT in plasma and BALF. (E-H) The concentrations of IL-6 and TNF-α in plasma and BALF [means ± standard deviation (SD); *n* = 10; * *P* < 0.05, ** *P* < 0.01].

The secretion of four cytokines in macrophages significantly increased after MRSA infection. Concentrations of C-reactive protein (CRP), procalcitonin (PCT), interleukin-6 (IL-6), and tumor necrosis factor-alpha (TNF-α) in plasma and bronchoalveolar lavage fluid (BALF) measured at 6 h after infection were notably higher than those at 54 h (Fig. 4). The levels of these cytokines decreased after treatments at 54 h. There were no significant differences in CRP and PCT between the antibiotics and antibiotic +LFU groups (Fig. 4A through D). However, the levels of IL-6 and TNF-α were lower in antibiotic-alone groups than those in their corresponding antibiotic + LFU groups, especially between the VCM +LFU group and the VCM-alone group (Fig. 4E through H).

### Relative expressions of MRSA-related and pneumonia-related genes

**Fig 5:**
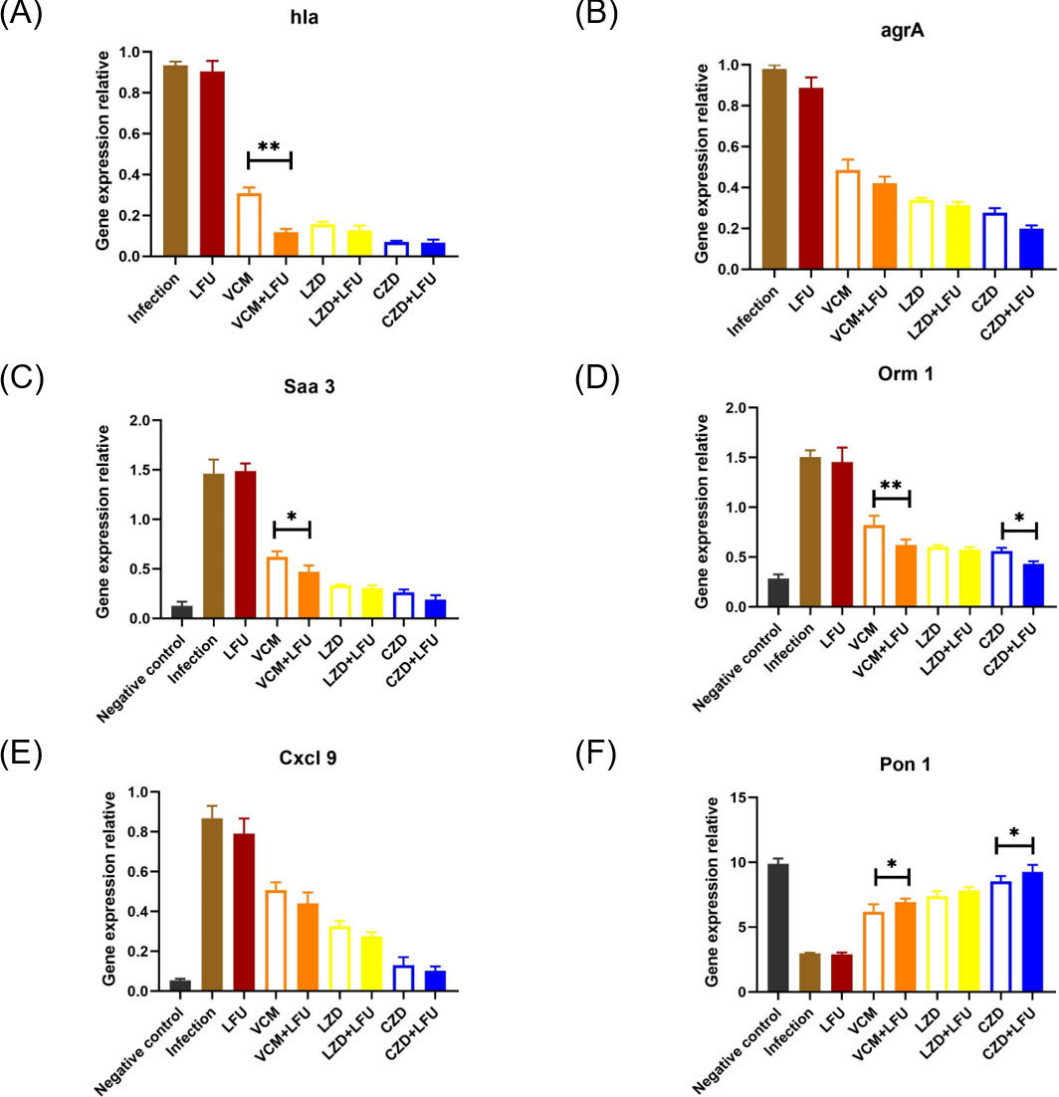
(**A and B**) Effects of antibiotic on the mRNA expression of hla and agrA at 54 h (6 h after the last treatment) after infection, as analyzed by quantitative reverse transcription-polymerase chain reaction(qRT-PCR). (**C–F**) Effects of antibiotics on mRNA expression after lung infection with MRSA at 54 h (6 h after the last treatment) after infection [means ± standard deviation (SD); *n* = 10; *P < 0.05; **P < 0.01].

Three antibiotics alone or in combination with LFU had inhibitory effects on the *hla* and *agrA* genes, with the CZD-containing regimen showing the most obvious effect (Fig. 5A and B). A significant inhibitory effect of the VCM and VCM + LFU groups on hla was also observed (Fig. 5A). Compared with their expressions in the negative control group, the *Saa3*, *Cxcl9*, and *Orm1* genes were significantly upregulated, while the *Pon1* gene was significantly downregulated in the lungs after MRSA infection (Fig. 5C through F). LFU alone did not show any effect on gene expression compared with the infection group. However, the expressions of *Saa3*, *Cxcl9*, and *Orm1* were decreased, and the expression of *Pon1* was increased after antibiotic treatments in the absence or presence of LFU. Moreover, the expressions of *Saa3* and *Orm1* of the VCM +LFU group were significantly lower, while the expression of *Pon1* was higher compared with that of the VCM-alone group.

### Antibiotic distribution in plasma and lungs

**Fig 6:**
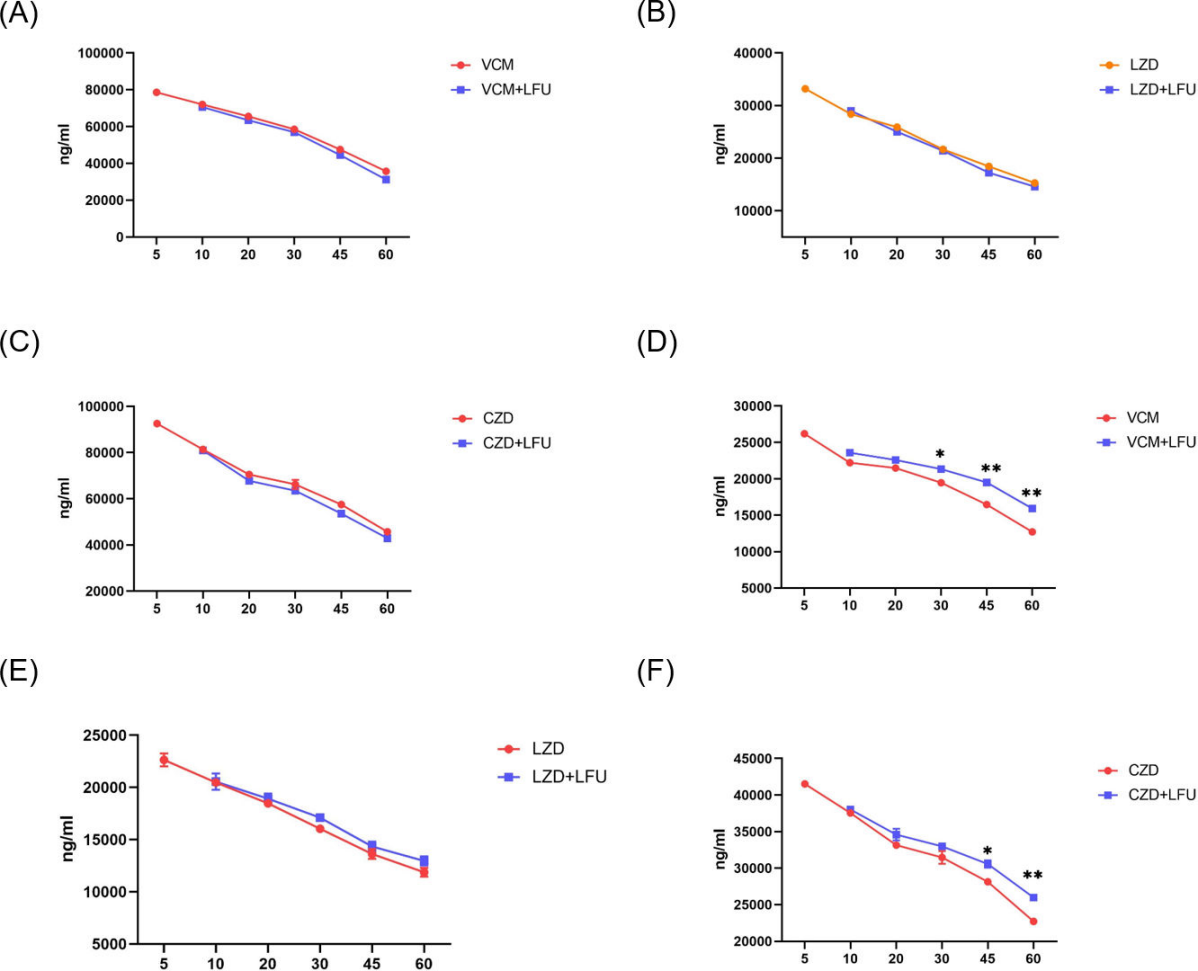
The concentration of antibiotics in plasma (**A–C**) and homogenate (**D–F**) [means ± standard deviation (SD); *n* = 6; *P < 0.05; **P < 0.01].

The concentrations of VCM, LZD, and CZD in the plasma were similar between the antibiotic-alone groups and their corresponding antibiotic +LFU groups (Fig. 6A through C). At 45 min, the concentration of CZD in lung tissue of the CZD +LFU group was higher compared with that of the CZD-alone group (Fig. 6F). The concentration of VCM in lung tissue was significantly increased when combined with LFU. From 30 min the VCM concentration of the VCM +LFU group was higher compared with that of the VCM group. At 60 min, the difference in tissue concentration between the two groups reached 3,215 ng/mL (Fig. 6D).

## DISCUSSION

As a safe and noninvasive method, LFU stimulates antibiotic transport through cells and biofilms via cavitation, high pressure, and high shear stress (23). In this *in vivo* study, we provided evidence that LFU improves the efficacy of VCM, LZD, and CZD in the MRSA pneumonia model. The results indicate that LFU not only promotes transdermal penetration of medicines but also enhances antibiotic distribution in mouse lung tissue. The entry of VCM and CZD from blood vessels into lung tissue was notably promoted, especially for VCM with low pulmonary penetration. This finding suggests that LFU can enhance medicine absorption through the epidermis and improve penetration in deep tissue at a certain frequency and intensity. VCM, considered the gold standard for MRSA pneumonia treatment, has variable lung penetration (24). A previous study investigated VCM penetration into lung tissue in patients undergoing partial lobectomy, revealing concentrations ranging from 0 to 12.2 mg/L, with a mean concentration of 2.8 mg/L and permeability of 41% (25). In our study, the ratio (lung/plasma) of VCM increased from 35% to 51% at 60 min after LFU treatment. Conversely, oral LZD at a dose of 600 mg showed a mean site/serum concentration ratio of 0.79 for bronchial mucosa, 0.71 for macrophages, and 8.35 for epithelial lining fluid (26). Under LFU treatment, the ratio (lung/plasma) of LZD slightly increased from 77% to 82% at 60 min. Due to limited pharmacokinetic studies on CZD in lung tissue, our data showed an increased ratio (lung/plasma) of CZD from 49% to 60% at 60 min after LFU treatment. Therefore, LFU could significantly increase the concentration of VCM and CZD in lung tissue, likely enhancing the bactericidal effect.

Tian et al. have reported that LFU promotes medicine penetration and improves the efficacy of intrapleural administration in treating malignant pleural effusion (27). Our previous research has demonstrated LFU enhancing the antibacterial activity of amikacin in lung tissue (22). The mechanism of LFU promoting antibiotic distribution involves not only the cavitation effect but also thermal and acoustic microstreaming effects (28). Dormant bacteria in biofilms may become more sensitive to antibiotics after LFU treatment (21). LFU’s wave nature allows it to be focused through tissue, generating energy that disrupts cell membrane structures, increasing vascular and alveolar permeability to improve antibiotic osmotic concentration (29). LFU’s thermal effect raises lung temperature, affecting membrane fluidity and enhancing antibiotic permeability (30). Acoustic microstreaming refers to LFU’s unidirectional liquid flow, driving antibiotic transmission from high concentration in blood vessels to low concentration in alveoli (31).

Histopathological results have demonstrated that LFU reduced inflammatory lung injury in mice, possibly due to LFU-induced cell proliferation and promotion of mesenchymal stem/progenitor cell differentiation (32). LFU affects the microenvironment around pneumonia, inducing immune activation, inhibiting inflammatory factors, increasing angiogenesis, and promoting inflammation repair (33). LFU enhances tissue cell proliferation, promoting inflammatory repair (34). Our study showed that LFU combined with antibiotics achieved the same antibacterial effect in mice as *in vitro* studies. LFU increased oxygen saturation, elevated hemoglobin concentration, and improved microcirculation at the inflammatory site (33). LFU reduced TNF-α and IL-6 secretion, as well as CRP and PCT protein production associated with acute inflammation. LFU’s immune modulation effects involve mechanical effects, ultrasonic pulses, or continuous thermal effects, leading to increased T-cell helper/suppressor ratio (35), dendritic cell infiltration, activated CTLs, NK cells, and downregulation of various inflammation-associated factors (36). Combining LFU with adoptive immunotherapy has been reported, indicating increased T-lymphocyte numbers, enhanced cytotoxicity, and elevated secretion of IFN-γ and TNF-α (37).

The *hla* gene, encoding α-hemolysin, is regulated by *agrA*, an essential component of the agr quorum-sensing system. This bacterial regulatory system detects extracellular self-inducers, controlling the virulence of various bacterial pathogens, including the regulation of α-hemolysin (38). In *S. aureus*, agr quorum sensing induces increased expressions of virulence genes, including those encoding toxins and degrading enzymes crucial for infection establishment (39). Our results indicate that VCM + LFU significantly reduced *hla* expression, suggesting that LFU enhanced antibacterial activity and inhibited MRSA virulence by reducing toxin production and related gene expression. *SAA3, CXCL9,* and *Orm1* are induced by pulmonary inflammation, while *Pon1* is associated with oxidative stress in sepsis caused by MRSA (40–43). Our findings showed that the LFU combination groups had decreased expressions of *Saa3*, *Cxcl9*, and *Orm1* compared with the corresponding antibiotic-alone groups, with increased *Pon1* expression. This suggested that LFU could mitigate lung tissue inflammation induced by pneumonia. Although the LFU group tended to have lower injury scores and cytokine expression than the infection group, no significant differences in gene expressions were observed between the LFU and infection groups. The lack of noticeable difference may be due to the low intensity and short duration of LFU treatment.

LFU, as a physically assisted anti-infection method, is primarily studied in *in vitro* and animal experiments, with clinical research focused on rehabilitation and chronic wound healing after epidermal debridement. LFU has shown effects such as reducing antibacterial drug usage, lowering infection recurrence rates, and promoting tissue regeneration. Studies demonstrate LFU’s ability to penetrate the human thorax and sternum, providing noninvasive monitoring of lung air and water content (44, 45). However, the translation of LFU effects from small animals to humans in the context of lung tissue infection requires further exploration. Safety is a primary concern in clinical applications, alongside efficacy evaluation. Variations in anatomy between animals and humans underscore the need for cautious extrapolation from animal experiments to clinical practice. Despite some understanding of LFU’s mechanism, there are significant discrepancies in frequency, intensity, and application time in *in vitro* research, necessitating further evaluation of parameters for clinical use. While the journey from theory to practice may be long, LFU holds great promise as a physical anti-infection method for addressing bacterial resistance and biofilm challenges in the future.

### Conclusions

As the pioneer of *in vivo* study exploring the combination of LFU with antibiotics for treating MRSA pneumonia, our findings revealed significant improvements in the survival rate of mice, accelerated clearance of pulmonary bacteria, reduced inflammatory injury, suppression of MRSA endotoxin production, and enhanced distribution of antibiotics in lung tissue when LFU was combined with VCM, LZD, and CZD. The application of LFU in the treatment of pulmonary infection holds profound significance and might open new avenues for therapeutic interventions.

## MATERIALS AND METHODS

### Strains, antibiotics, and antimicrobial susceptibility testing

The study utilized MRSA 159228, clinically isolated from the Chinese PLA General Hospital, with ATCC 29213 serving as a quality control strain for MIC determination (46). VCM and LZD were sourced from Macklin (Shanghai, China), while CZD was provided by MicuRx (Shanghai, China). MICs were determined using the broth microdilution method, following Clinical and Laboratory and Laboratory Standards Institute guidelines (47). Concentrations of antibiotics in 96-well plates ranged from 0 to 128 µg/mL, with the MIC determination repeated thrice. MRSA 159228 and ATCC 29213 were cultured on Mueller-Hinton agar (MHA) and then suspended in Mueller-Hinton broth. The resulting bacterial suspension (1 × 10^6^ CFU/mL) was incubated at 37°C for 24 h.

### Animals, mouse pneumonia model, and drug distribution model

Institute of Cancer Research (ICR) mice (male, 6–8 weeks old, weighing 20–25 g) from the PLA General Hospital animal facility were used. Animal experiments received approval from the Chinese PLA General Hospital’s animal ethics committee (SQ2022423). Mice were housed in a pathogen-free environment with controlled temperature and light conditions and provided sterile feed and water.

The lung infection model involved intratracheal instillation of 100 µL of MRSA 159228 suspension (9 × 10^8^ CFU/mL) after anesthesia (48). The drug distribution model utilized tail vein injections of VCM (100 mg/kg), LZD (80 mg/kg), and CZD (120 mg/kg) based on body weight. A total of 198 healthy ICR mice were divided into six groups, three treated with antibiotics alone and three with LFU in combination with antibiotics. LFU was applied immediately after each antibiotic administration for 10 min. Plasma and lung tissue homogenates were sampled at designated time intervals. Sampling times were determined based on peak plasma concentrations and the lower limit of quantitation.

### Experimental sampling and group details

Sampling times included 5, 10, 20, 30, 45, and 60 min after administration in the antibiotic-alone groups and 10, 20, 30, 45, and 60 min in the LFU combination groups. Six mice were assigned to each group at each time point, except for the LFU-combined antibiotic group at 5 min. The experimental design considered the peak time of VCM, LZD, and CZD plasma concentrations and the lower limit of quantitation (49–51). After 1 h, drug concentrations in mouse lung tissue were extremely low. Table 1 provides a summary of the experimental groups and sampling details.

**TABLE 1:** Experimental grouping of animal models of drug distribution

Group	Time (min)						Total
	5	10	20	30	45	60	
VCM	*n* = 6	*n* = 6	*n* = 6	*n* = 6	*n* = 6	*n* = 6	36
VCM + LFU	-	*n* = 6	*n* = 6	*n* = 6	*n* = 6	*n* = 6	30
LZD	*n* = 6	*n* = 6	*n* = 6	*n* = 6	*n* = 6	*n* = 6	36
LZD + LFU	-	*n* = 6	*n* = 6	*n* = 6	*n* = 6	*n* = 6	30
CZD	*n* = 6	*n* = 6	*n* = 6	*n* = 6	*n* = 6	*n* = 6	36
CZD + LFU	-	*n* = 6	*n* = 6	*n* = 6	*n* = 6	*n* = 6	30

### LFU apparatus and treatment protocol

The LFU apparatus was provided by Beijing Nava Medical Technology. The fixed working frequency was 29.36 kHz, and the effective output intensity ranged from 250 to 300 mW/cm². In this study, the LFU operated at 270 mW/cm² for 10 min, a parameter determined based on the degree of burn observed on mouse skin under different output power levels and durations in our preliminary experiments.

The effective working diameter of the ultrasonic tool head was 1.5 cm, and the working mode was pulse modulation. Mice were initially anesthetized in an induction box, and then their mouths and noses were placed in a continuous anesthesia mask. While continuously inhaling isoflurane, mice were positioned in the supine position, their limbs were extended, and the chest skin was fully exposed. The limbs were secured on a multifunctional small-animal experimental console at an adjustable angle with a medical tape. A sufficient amount of ultrasonic couplant was applied to the chest skin of the mice, and the working surface of the LFU probe was adjusted to snugly fit the mouse’s chest without compression. The mice were divided into nine groups in the mouse pneumonia model, each consisting of 10 mice: the negative control group, infection group, LFU group, VCM group, VCM +LFU group, LZD group, LZD + LFU group, CZD group, and CZD +LFU group. The negative control group was inoculated with saline, while the other groups received MRSA 159228 suspension. At 12 h postinfection, VCM (100 mg/kg, q.12h), LZD (80 mg/kg, q.12h), and CZD (120 mg/kg, q.12h) were administered via intraperitoneal injection.

**Fig 7:**
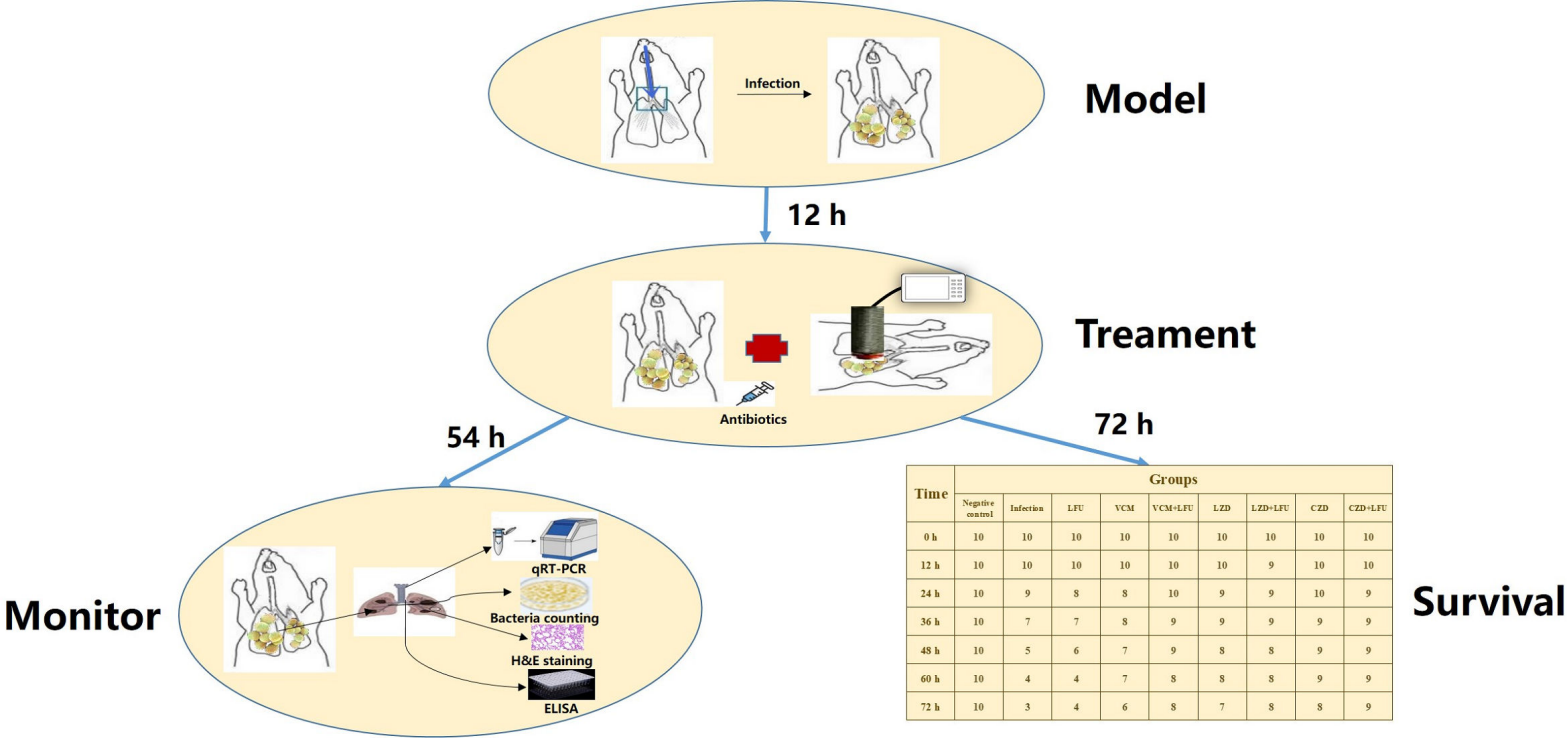
Schematic diagram of the mice experiment process.

Mice in the infection group were treated with 100 µL of sterile normal saline (NS) with the same schedule as the solvent control. The dosages of VCM and LZD were determined based on recommended human dosages of 15 mg/kg for VCM and 10 mg/kg for LZD (52). CZD was administered at a dose of 120 mg/kg, equivalent to 800 mg/kg of human body weight, to assess its therapeutic potential against MRSA (53, 54). LFU was applied immediately for 10 min after each antibiotic administration. The experimental procedure is illustrated in Fig. 7.

The infection and LFU groups were treated 12 h postinfection and intraperitoneally injected with the same amount of NS. Mice in the LFU group received LFU for 10 min after each NS injection, twice a day for two consecutive days. LFU was not performed in the infection group. The negative control group received no medication or LFU treatment, except for saline inoculation.

Mice in each group were sacrificed at 54 h after the last inoculation (6 h). This time point was chosen considering the expected 50% survival rate of mice in the infected group at 48 h. Additionally, 90 extra mice were grouped and treated as previously to observe the survival rate up to 72 h after infection (Fig. 7).

### Lung bacterial clearance assay

After the blood and BALF samples were collected, the right lungs of the mice were dissected and homogenized in sterile phosphate-buffered saline (PBS) (1 mL: 100 mg). Serial dilutions (1:10) were prepared in PBS and inoculated on MHA plates followed by incubation at 37°C for 24 h. Subsequently, the bacterial colonies were counted, and the colony count formation of the lung homogenate was calculated and expressed as CFU/g.

### Histological sample preparation and histopathological injury scores

The left lung of mice was fixed in 10% neutral-buffered formalin for 24 h, embedded in paraffin, and then cut into 5-µm-thick sections. Subsequently, these sections underwent staining with H&E and were observed under an optical microscope. To generate a lung injury score, 300 alveoli on each slide were counted at a magnification of ×400. Within each area, scores were assigned based on predetermined criteria.

Lung injury was assessed considering four aspects: (i) alveolar septal congestion (ii), bleeding (iii), aggregation or infiltration of neutrophils into the alveoli, and (iv) fibrin chains in the alveoli. Each criterion was scored on a four-point scale: 3 for maximum injury, 2 for moderate injury, 1 for slight damage, and 0 for minimum (or no) damage. The damage score was calculated using the following formula: injury score = [(alveolar hemorrhage points/no. of fields) + 2 × (alveolar infiltrate points/no. of fields) + 3 × (fibrin points/no. of fields) + (alveolar septal congestion/no. of fields)]/total number of alveoli counted (55).

### Cytokine measurement in plasma and BALF

In accordance with previously published research, cytokine expressions peak at 6 h post-MRSA inoculation and subsequently decrease (56). At the 6-h time point, which is 54 h after inoculation, blood samples were obtained via eye extraction, and BALF was collected through intratracheal injection of 1 mL of sterilized PBS into the lungs, followed by immediate vacuum suction (57). These samples underwent centrifugation at 3,500 rpm/min for 10 min at 4°C. The resulting supernatant was collected and stored at −80°C until enzyme-linked immunosorbent assay (ELISA).

Concentrations of CRP, PCT, IL-6, and TNF-α in both plasma and BALF were determined using specific mouse ELISA kits (MlBio, Shanghai, China), following the manufacturer’s instructions. Data analysis involved curve fitting to the standard, and sample concentrations were extrapolated from the standard curve using the four-parameter logic software.

### RNA extraction and quantitative reverse transcription-polymerase chain reaction

In addition to investigating the impact of inflammation-related genes, we examined the effects of LFU on gene expression in lung tissue, focusing on genes encoding serum amyloid A3 (*Saa3*), chemokine (C-X-C motif) ligand 9 (*Cxcl9*), orosomucoid 1 (*Orm1*), and paraoxonase 1 (*Pon1*). Additionally, we assessed the influence of LFU on the mRNA expression of MRSA virulence factors, including α-hemolysin (*hla*) and accessory gene regulator (*agrA*). These factors are pivotal in the pathogenesis of MRSA infection and are commonly employed in *S. aureus* strain studies (58). For internal references, we selected standardized genes commonly used in MRSA studies, namely, 16S rRNA and GAPDH, around the quantitative reverse transcription (qRT) process.

Total RNA was extracted from homogenized lung tissue using the Direct-zol RNA MiniPrep Plus total RNA extraction kit (Zymo Research, USA), following the manufacturer’s instructions. The dissolved total RNA in diethyl pyrocarbonate-treated distilled water was assessed for purity using a spectrophotometer (BioTek, USA), measuring the absorbance ratio between 260 and 280 nm. Complementary DNA was synthesized with the QuantiTect reverse transcription kit (Biotechnology, Inc., New England), as per the manufacturer’s instructions. qRT-PCR was conducted in triplicate using PowerUp SYBR Green PCR master mix (Applied Biosystems, USA) on a StepOnePlus real-time PCR system (Applied Biosystems). Following an initial denaturation step at 95°C for 10 min, the amplifications comprised 40 cycles at a melting temperature of 95°C for 15 s and an annealing temperature of 60°C for 1 min. The relative expressions of target genes were normalized to the endogenous reference gene, β-actin, and calculated using the 2-ΔΔCt method with StepOne software v2.3 (Applied Biosystems). The primer sequences are provided in Table S1.

### Determination of antibiotics in plasma and lung homogenates by liquid chromatography coupled with mass spectrometry

The concentrations of VCM, LZD, and CZD in plasma and lung homogenates were determined by high-performance liquid chromatography coupled with mass spectrometry (Agilent Technologies 3000, USA). All sample processing and thawing of frozen plasma samples were performed at 4°C. The specific detection methods of VCM, LZD, and CZD are shown in Table S2. After verification, these methods were stable and effective.

### Statistical analysis

Data were expressed as mean ± standard deviation. Ordinary one-way analysis of variance was used to analyze CFU (colony-forming units), histopathological scores, cytokine concentrations, and relative gene expression. Additionally, the Holmes Sidak-corrected multiple *t*-test was employed to evaluate antibiotic concentrations in plasma and lung homogenates. GraphPad Prism software version 8.0 (GraphPad Software, San Diego, CA, USA) was used for statistical analysis. *P* values <0.05 were considered statistically significant. The nucleotide sequence accession number [National Center for Biotechnology Information (NCBI)] of each gene is designed and generated through the NCBI website. We determined the sample size of mice used in our experiment based on a pre-experiment using the KISS determination method (59).

## Data Availability

The data that support the findings of this study are available from the corresponding author upon reasonable request.

## SUPPLEMENTAL MATERIAL

Supplemental tables spectrum.01016-23-s0001.docx Tables S1 and S2.

ASM does not own the copyrights to Supplemental Material that may be linked to, or accessed through, an article. The authors have granted ASM a non-exclusive, world-wide license to publish the Supplemental Material files. Please contact the corresponding author directly for reuse.
