# Supplementary material for: Effects of low-frequency ultrasound combined with anti-MRSA agents on the mouse model of pulmonary infection
Source: Microbiol Spectr. 2024 Feb 7;12(3):e01016-23. doi: 10.1128/spectrum.01016-23 (PMC10913739; doi:10.1128/spectrum.01016-23)
Supplement: Supplemental tables — Tables S1 and S2. [file spectrum.01016-23-s0001.docx]

**Supplemental Table 1. Sequences of oligonucleotide primers designed for real-time PCR**

| Gene |  | Primer sequence | NCBI |
| --- | --- | --- | --- |
| Saa3 |  |  | NM_011315.3 |
|  | Forwd | GCCTGGGCTGCTAAAGTCAT |  |
|  | Revere | TGCTCCATGTCCCGTGAAC |  |
| Orm1 |  |  | NM_008768.2 |
|  | Forwd | GTGTGTCTATAACTCCACCCATC |  |
|  | Revere | CCCATGTTTCCTCAGCACTAT |  |
| CXCL9 |  |  | NM_008599.4 |
|  | Forwd | GGCACGATCCACTACAAATCC |  |
|  | Revere | GGTTTGATCTCCGTTCTTCAGT |  |
| Pon1 |  |  | NM_001410259.1 |
|  | Forwd | GGACTAACTTTCTTTAGCATGGC |  |
|  | Revere | TTCTAACTCTGACACTGCTGGCC |  |
| GAPDH |  |  | NM_001256799.3 |
|  | Forwd | TGGGCTACACTGAGCACCAG |  |
|  | Revere | GGGTGTCGCTGTTGAAGTCA |  |
| *hla* |  |  | NC_007795.1 |
|  | Forwd | TTGGTGCAAATGTTTC |  |
|  | Revere | TCACTTTCCAGCCTACT |  |
| agrA |  |  |  |
|  | Forwd | TGATAATCCTTATGAGGTGC | NC_000913.3 |
|  | Revere | CACTGTGACTCGTAACGAAA |  |
| 16S rRNA |  |  | NR_118997.2 |
|  | Forwd | CGTGCTACAATGGACAATAC |  |
|  | Revere | ATCTACGATTACTAGCGATT |  |

NCBI: the nucleotide sequence accession number

**Supplemental Table 2. Chromatographic and mass spectrometric conditions, preparation of the standard solution, and pretreatment of VCM, LZD, and CZD**

| **Methods** | | **Vancomycin** | **Linezolid** | **Contezolid** |
| --- | --- | --- | --- | --- |
| **Chromatographic Conditions** | **Chromatographic Column** | Eclipse Plus C8 (2.1x100mm, 3.5μm) | Eclipse Plus C18 (2.1x100mm, 3.5μm) | Eclipse Plus C18 (2.1x100mm, 3.5μm) |
|  | **Mobile Phase** | A: 0.1% formic acid in 2 mM ammonium acetate; B: Methanol | A: 0.1% formic acid in 10 mM ammonium acetate solution; B: 0.1% formic acid in 5 mM ammonium acetate in acetonitrile water (90:10, v/v) | A: 0.1% formic acid in 10 mM ammonium acetate solution; B: 0.1% formic acid in 5 mM ammonium acetate in acetonitrile water (90:10, v/v) |
|  | **Elution Gradient** | 0~0.2min:90%A，10%B;  0.2~1.5min:5%A，95%B; 1.5~2.5min:5%A，95%B;  2.5~2.6min:90%A，10%B; 2.6~4min:90%A，10%B; | 0~0.5min:85%A，15%B;  0.5~1.6min:85%A，15%B; 1.6~4min:70%A，30%B;  4~5min:50%A，50%B; 5~5.1min:50%A，50%B; 5.1~6min:85%A，15%B； | 0~0.5min:85%A，15%B;  0.5~1.6min:85%A，15%B; 1.6~4min:70%A，30%B;  4~5min:50%A，50%B; 5~5.1min:50%A，50%B; 5.1~6min:85%A，15%B； |
|  | **Current Speed** | 0.4 mL/min | 0.4 mL/min | 0.4 mL/min |
|  | **Column Temperature** | 40℃ | Room temperature | Room temperature |
|  | **Injection volume** | 5 μl | 5 μl | 5 μl |
|  | **Internal standard** | Contezolid | Contezolid | Linezolid |
| **Mass-spectrum Conditions** | **Ionization Mode** | Electrospray ionization source | Electrospray ionization source | Electrospray ionization source |
|  | **Measured object ion pair** | 724.7/144；724.7/99.6 | 338.14/195.1 | 409.15/269.14 |
|  | **Internal standard ion pair** | 409.15/269.14；409.15/122.8 | 409.15/269.14 | 338.14/195.1 |
| **Solution Preparation** | **Standard** | 50,000, 40,000, 20,000, 10,000, 5,000, 2,500, 1,000, 500 ng/mL | 15,000, 12,000, 8,000, 5,000, 3,000, 1,500, 800, 400 ng/mL | 5,000, 4,000, 2,500, 1,000, 500, 250, 100, 50 ng/mL |
|  | **Quality control** | 37,500, 15,000, 1,500, 500 ng/mL | 10,000, 4,000, 1,200, 400 ng/mL | 3,750, 2,000, 150, 50 ng/mL |
|  | **Internal standard** | 40 ng/mL | 2,000 ng/mL | 800 ng/mL |
| **Sample Handling** | **Preparation of medicated plasma (homogenate)** | 10 μl vancomycin+90 μl plasma | 10 μl linezolid+90 μl plasma | 10 μl contezolid +190 μl plasma |
|  | **Pretreatment** | 50 μl drug-containing plasma + 30 μl 25% trichloroacetic acid + 200 μl contezolid (acetonitrile: methanol = 1:1) | 50 μl drug-containing plasma + 200 μl contezolid (acetonitrile: methanol = 1:1) | 50 μl drug-containing plasma + 250 μl linezolid (acetonitrile: methanol = 1:1) |
| **Retention Time** | **Target object** | 2.04 min | 3.83 min | 4.55 min |
|  | **Internal standard** | 2.52 min | 4.55 min | 3.81 min |
